# Mitochondrial RNA cytosolic leakage drives the SASP

**DOI:** 10.21203/rs.3.rs-4876596/v1

**Published:** 2024-08-27

**Authors:** Stella Victorelli, Madeline Eppard, Seung-Hwa Woo, Stacia P.A. Everts, Helene Martini, Nicholas Pirius, Ana Catarina Franco, Yeaeun Han, Dominik Saul, Patrick L. Splinter, Steven P. O’Hara, Lucía Valenzuela-Pérez, Hyun Se Kim Lee, Diana Jurk, Nicholas F. LaRusso, Petra Hirsova, João F. Passos

**Affiliations:** 1 Department of Physiology and Biomedical Engineering, Mayo Clinic, Rochester, MN 55905, USA; 2 Robert and Arlene Kogod Center on Aging, Mayo Clinic, 200 First Street SW, Rochester, MN 55905, USA; 3 Division of Gastroenterology and Hepatology, Mayo Clinic, Rochester, MN, USA.

## Abstract

Senescent cells secrete proinflammatory factors known as the senescence-associated secretory phenotype (SASP), contributing to tissue dysfunction and aging. Mitochondrial dysfunction is a key feature of senescence, influencing SASP via mitochondrial DNA (mtDNA) release and cGAS/STING pathway activation. Here, we demonstrate that mitochondrial RNA (mtRNA) also accumulates in the cytosol of senescent cells, activating RNA sensors RIG-I and MDA5, leading to MAVS aggregation and SASP induction. Inhibition of these RNA sensors significantly reduces SASP factors. Furthermore, BAX and BAK plays a key role in mtRNA leakage during senescence, and their deletion diminishes SASP expression *in vitro* and in a mouse model of Metabolic Dysfunction Associated Steatohepatitis (MASH). These findings highlight mtRNA’s role in SASP regulation and its potential as a therapeutic target for mitigating age-related inflammation.

## Introduction

Cellular senescence is an irreversible growth arrest triggered by stressors, involving activation of p53/p21^CIP1^ and p16^INK4A/RB^ pathways^1–3^. Senescent cells secrete proinflammatory cytokines, chemokines, and extra-cellular matrix degrading proteins, which are collectively known as the senescence-associated secretory phenotype (SASP)^4,5^. Chronic exposure to the SASP can lead to tissue dysfunction^6^. Furthermore, senescence has been shown to occur in multiple tissues during aging and in multiple chronic diseases^7–10^. Notably, clearance of senescent cells has been shown to improve several age-related conditions^11–13^.

Mitochondrial dysfunction is a key feature of cellular senescence. To functionally investigate the role of mitochondria in cellular senescence we utilized the property of experimentally induced PINK1/Parkin to induce widespread mitophagy to generate human fibroblasts without mitochondria^14^. Senescent cells with no mitochondria had no SASP, but still underwent the irreversible cell cycle arrest ^15,16^. This led us to propose that mitochondria play a key role in the regulation of the SASP and may be promising targets for anti-senescence therapies that suppress the detrimental SASP, while maintaining the tumor suppressor capabilities of senescent cells^15,16^. We recently found that mitochondria influence the SASP by releasing mitochondrial DNA (mtDNA) into the cytosol, activating the cGAS/STING pathway^17^.

Mitochondrial RNA (mtRNA) is highly immunogenic likely due to the absence of nucleoside modifications^18^. Abnormal accumulation of endogenous mitochondrial double-stranded RNA (mtdsRNA) in the cytosol has been shown to trigger antiviral signaling and induce a type I interferon response, leading to inflammation^19^. Recent studies have demonstrated that RNA viruses, such as SARS-CoV-2, can trigger senescence and induce the SASP^20,21^. Although viral RNA is known to accelerate senescence, whether endogenous mtRNA can leak into the cytosol of senescent cells and activate pro-inflammatory phenotypes is unknown.

In this study we sought to investigate the role of mtdsRNA in the regulation of the SASP during cellular senescence. We found that senescent cells accumulate cytosolic mtdsRNA, which activates RNA sensors RIG-I and MDA-5, leading to Mitochondrial Antiviral Signaling protein (MAVS) aggregation. Inhibiting RIG-I, MDA-5, and MAVS suppresses the SASP, highlighting the role of mtdsRNA in SASP regulation and its potential as a therapeutic target. Additionally, we discovered that mtdsRNA leakage in senescent cells depends on BAX and BAK. Consistently, deleting BAX and BAK in a mouse model of Metabolic Dysfunction Associated Steatohepatitis (MASH) reduced expression of RNA sensors and SASP factors.

## Results:

### Senescent cells have increased cytosolic mtdsRNA and RNA sensor signaling

**Figure 1:**
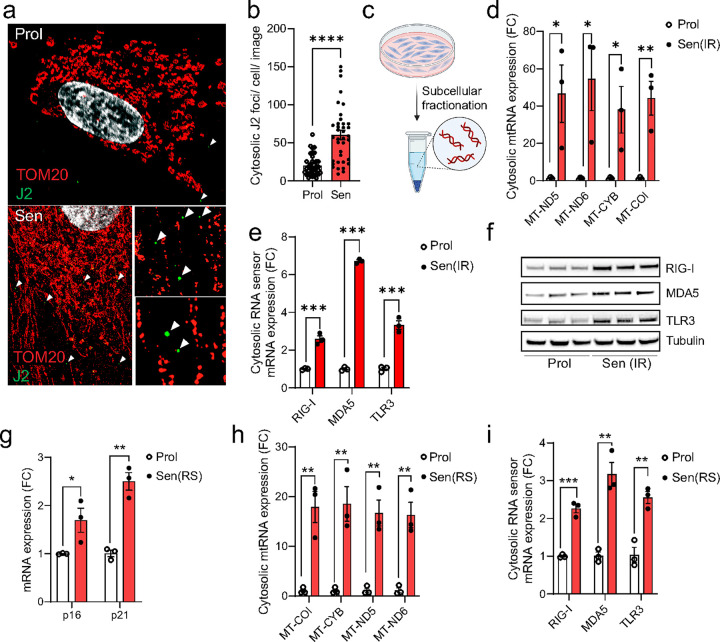
Cytosolic mtRNA leakage is a feature of senescent cells. (a) Representative super-resolution Airyscan microscopy image of TOM20 (red) and J2 (dsRNA; green) in proliferating and senescent (IR) MRC5 human fibroblasts showing that dsRNA foci are enriched in the cytoplasm of senescent cells. (b) The number of cytosolic dsRNA foci in the cytosol of proliferating and senescent (IR) MRC5 human fibroblasts. (c) Representative scheme of subcellular fractionation method. (d, e) qPCR quantification of (d) cytosolic mtRNA transcripts and (e) cytosolic RNA sensors in proliferating and senescent (IR) MRC5 human fibroblasts. n=3 independent experiments. (f) Western blot showing expression of RNA sensors RIG-I, MDA5, and TLR3 in proliferating and senescent (IR) MRC5 human fibroblasts. n=3 independent experiments. (g) mRNA levels of senescence markers p16 and p21 in proliferating and senescent (IR) MRC5 human fibroblasts. (h, i) qPCR quantification of (h) cytosolic mtRNA transcripts and (i) cytosolic RNA sensors in proliferating and senescent (IR) MRC5 human fibroblasts. n=3 independent experiments. Data are mean ± s.e.m. Statistical significance was assessed by a two-sided Student’s unpaired t-test. *p<0.05, **p<0.01, ***p<0.001, ****p<0.0001.

Using dual immunostaining of the mitochondria outer membrane protein TOM20 and J2 antibody, which recognizes double stranded RNA (dsRNA), visualized by super-resolution Airyscan confocal microscopy, we observed an increased frequency of dsRNA nucleoids in the cytosol of senescent fibroblasts (Figure 1a & b). Subsequent subcellular fractionation of senescent cells (Figure 1c) revealed elevated levels of mitochondrial RNA in the cytosolic fraction. Specifically, there was an increase in cytosolic mitochondrial-derived RNA transcripts, including MT-ND5, MTND6, and MT-CYB and MT-COI (Figure 1d). This rise in cytosolic mitochondrial-derived RNA was accompanied by a significant upregulation of cytosolic RNA sensors RIG-I, MDA5, and TLR3 at both the mRNA and protein levels in senescent compared to proliferating cells (Figure 1e & f). These findings were consistent irrespective of the senescence-inducing stimulus, including in replicative senescent cells (Figure 1g–i) and cells induced to senescence by the chemotherapy drugs doxorubicin and etoposide (**Extended Data Figure 1a-e**), and in a different cell line (IMR90 fibroblasts) (**Extended Data Figure 1f-h**).

Furthermore, we observed that expression of RNA sensors Rig-I, Mda-5 and Tlr3 significantly increased with age in murine kidney, heart, liver and spleen, concommitantly with increased expression of senescence-associated markers p16 and p21. Notably, across these tissues, RNA sensor expression exhibited a positive correlation with the expression of p21, p16, and several SASP factors (**Extended Data Figure 2a-t**). These findings suggest a mechanistic link between cellular senescence, the upregulation of RNA sensors, and the SASP.

### Cytosolic mtdsRNA is a driver of the SASP

**Figure 2:**
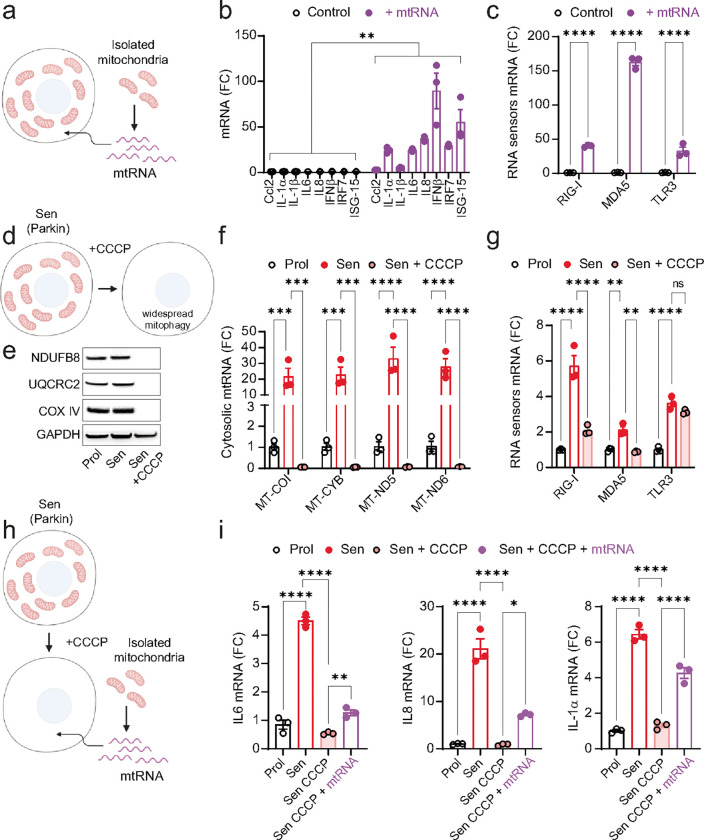
Cytosolic mtRNA is a driver of the SASP. (a) Scheme showing the isolation of mitochondrial RNA (mtRNA) and its subsequent transfection into cells. (b, c) qPCR quantification of (b) SASP factors and (c) RNA sensors in proliferating MRC5 fibroblasts and following transfection with mtRNA. n=3 independent experiments. (d) Scheme representing Parkin-mediated widespread mitophagy following CCCP treatment. (e) Western blot showing the expression levels of mitochondrial proteins NDUFB8, UQCRC2 and COX IV, demonstrating the absence of mitochondrial proteins following Parkin-mediated clearance. (f, g) qPCR quantification of (f) cytosolic mtRNA genes MT-COI, MT-CYB, MT-ND5, MT-ND6 and (g) RNA sensors in Parkin-expressing IMR90 fibroblasts after widespread mitophagy. n=3 independent experiments. (h) Experimental scheme showing senescent Parkin-expressing IMR90 fibroblasts being transfected with mtRNA following Parkin-mediated widespread mitophagy. (i) mRNA expression of SASP genes in senescent Parkin-expressing IMR90 fibroblasts following widespread mitophagy and after transfection with mtRNA. n=3 independent experiments. Statistical significance was assessed by a two-sided Student’s unpaired t-test (b, c) and a oneway ANOVA followed by Tukey’s multiple comparison test (f, g, i). *p<0.05, **p<0.01, ***p<0.001, ****p<0.0001.

Having observed an increase in cytosolic mtdsRNA in senescent cells, we investigated whether mtdsRNA alone is sufficient to drive the SASP. To this end, we transfected proliferating human fibroblasts with mtRNA and found a significant rise in expression of common SASP factors, along with elevated levels of RNA sensors MDA-5, RIG-I, and TLR3 (Figure 2a–c).

To further explore the role of mtRNA in inducing the SASP in senescent cells, we created cells without mitochondria to study the role of mtRNA in SASP regulation independently of other mitochondrial functions^14^. We stably transduced human fibroblasts with YFP-Parkin and induced senescence via X-ray irradiation (Figure 2d). Parkin-expressing senescent cells were treated with the mitochondrial uncoupler CCCP, triggering widepread mitophagy and generating mitochondria-free cells^14^. Western blot analysis confirmed the absence of mitochondrial proteins NDUFB8, UQCRC2, and COXIV following Parkin-mediated mitophagy (Figure 2e). qPCR analysis showed that cytosolic mtRNA transcripts were abrogated in these mitochondria-devoid senescent cells (Figure 2f), with a significant reduction in expression of RNA sensors MDA-5 and RIG-I and SASP factors (Figure 2g & i). When mitochondria-free senescent cells were transfected with mtRNA, the mRNA expression of SASP factors IL1-α, IL-6, and IL-8 was partially restored (Figure 2h–i) suggesting that mtRNA alone could drive the SASP.

Next, we sought to prevent mtRNA leakage during senescence by treating cells with an inhibitor of mitochondrial RNA polymerase (POLRMT), the enzyme responsible for transcribing mtDNA into RNA within mitochondria. Treating senescent cells with POLRMT inhibitor IMT1 significantly reduced cytosolic mtdsRNA levels (**Extended Data Figure 3a**) and the expression of RNA sensors RIG-I, MDA-5, TLR3 (**Extended Data Figure 3b**), and SASP components (**Extended Data Figure 2c-f**), without impacting on p21 and p16 expression (**Extended Data Figure 3g**). Collectively, these findings support mtdsRNA as a key driver of the SASP.

### RNA sensing signaling contributes to the development of the SASP

Having established that cytosolic mtRNA can contribute to the SASP, we sought to investigate the underlying mechanisms. Cytosolic RNA sensors are crucial components of the innate immune system, responsible for detecting dsRNA, which is often a sign of viral infection. These sensors ultimately converge into pro-inflammatory signaling pathways, such as NF-κB, interferon-regulatory factors (IRFs), and the NLRP3 inflammasome, leading to the induction of type-I interferon genes and pro-inflammatory cytokines^22–24^.

**Figure 3:**
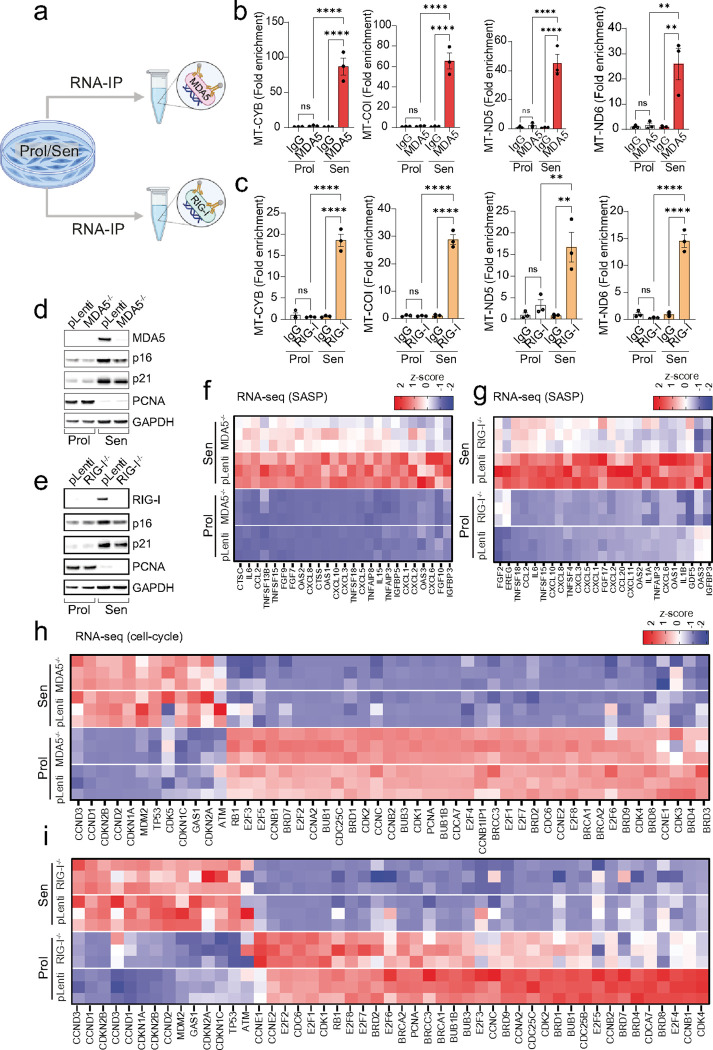
Cytosolic RNA sensors MDA5 and RIG-I are regulators of the SASP. (a) Scheme representing experimental design for RNA immunoprecipitation (RIP). (b, c) qPCR quantification of mtRNA genes following RNA-IP of (b) MDA5 and (c) RIG-I in proliferating and senescent cells. (d, e) Western blot showing the successful deletion of (d) MDA5 and (e) RIG-I, as well as expression of senescence markers p16, p21 and PCNA in proliferating and senescent fibroblasts. Representative of n=3 independent experiments. (f, g) Column-clustered heatmap of SASP genes that are upregulated in senescence and significantly downregulated upon (f) MDA5 and (g) RIG-I deletion. The color intensity represents the column z-score. (h, i) Column-clustered heatmap of cell-cycle related genes that are differentially expressed in senescent cells and not changed by (h) MDA5 and (i) RIG-I deletion. Color intensity represents the z-score. n=3 independent experiments. Statistical significance was assessed by a one-way ANOVA followed by Tukey’s multiple comparison test (b, c).

To investigate the role of RNA sensors in cellular senescence and their impact on the SASP, we first treated senescent cells with a TLR3/dsRNA complex inhibitor. However, this treatment did not affect the expression of common SASP components or cyclin-dependent kinase inhibitors p15, p16 and p21 (**Extended data Figure 4a-d**). Next, we immunoprecipitated the RNA sensors RIG-I and MDA-5 in both proliferating and senescent cells and performed q-PCR to detect mtRNA transcripts. Our results showed that RIG-I and MDA-5 in senescent cells exhibited increased interaction with mtRNA compared to proliferating cells (Figure 3 a–c).

Having observed the binding of RIG-I and MDA-5 to mtRNA in senescent cells, we utilized CRISPR/Cas9 gene editing to generate human fibroblasts deficient in RIG-I and MDA-5 (Figure 3d & e). RNA-sequencing revealed that the deletion of RIG-I or MDA-5 significantly reduced the expression of several SASP components (Figure 3f and g). Additionally, the deletion of RIG-I and MDA-5 resulted in a negative enrichment score for established senescence-associated gene sets, SenMayo^25^ and SenSig^26^, which encompass many SASP factors (**Extended data Figure 5 a-f**). It also significantly downregulated Gene Ontology (GO) terms related to inflammatory processes (**Extended Data Fig. 5g & h**) and reduced expression of several interferon-related genes (**Extended Data Fig. 5i & j**).

No change in expression of PCNA, p21 and p16 protein (Figure 3d & e) or mRNA proliferation genes normally downregulated in senescent cells was observed (Figure 3h & i), suggesting that RIG-I and MDA-5 regulate the SASP, but not the senescence-associated cell-cycle arrest. Together, these data suggest that RIG-I and MDA-5 recognize cytosolic mtRNA in senescent cells and play a role in SASP regulation.

### MAVS aggregation contributes to the SASP

Upon binding to RNA, MDA-5 and RIG-I oligomerize and interact with the adaptor protein MAVS (Mitochondrial Antiviral Signaling protein). MAVS oligomerization is a nucleation process where multiple MAVS molecules come together to form a large, functional aggregate ^27^. The aggregation of MAVS is essential for activation of transcription factors such as IRF3 and NF-κB which play key roles in SASP regulation^28^.

**Figure 4:**
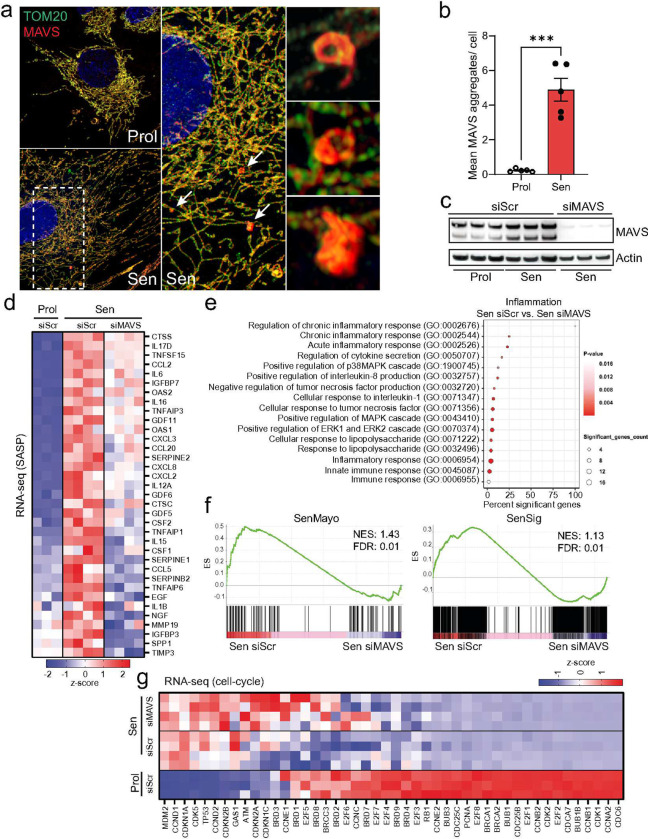
Aggregation of MAVS is a feature of senescent cells and plays a role in SASP regulation. (a) Representative super-resolution Airyscan microscopy image of TOM20 (green) and MAVS (red) in proliferating and senescent (IR) MRC5 fibroblasts showing MAVS aggregation in senescent cells. Arrows indicate MAVS aggregates that are amplified on the right. (b) The mean number of MAVS aggregates observed in proliferating and senescent MRC5 human fibroblasts. n=5 independent experiments. (c) Western blot showing successful small interfering RNA (siRNA) knockdown of MAVS in senescent MRC5 human fibroblasts. n=3 independent experiments. (d) Column-clustered heatmap of SASP genes upregulated in senescent cells and significantly downregulated upon knockdown of MAVS. Color intensity represents the z-score. n=3–4 independent experiments. (e) Gene ontology (GO) term enrichment analysis showing pathways related to inflammation that are significantly altered between senescent control (Sen siScr) and senescent cells lacking MAVS (Sen siMAVS). (f) The GSEA plots for Sen siScr and Sen siMAVS show an enrichment of SenMayo and SenSign genes in senescent control cells. (g) Columnclustered heatmap of cell-cycle genes upregulated in senescent cells and not changed by MAVS siRNA knockdown. Color intensity represents the column z-score. n=3–4 independent experiments. Statistical significance was assessed by a two-sided Student’s unpaired t-test (b). ***p<0.001.

To investigate MAVS aggregation in senescence, we performed dual immunostaining and visualized the mitochondrial outer membrane protein TOM20 and MAVS using Airyscan microscopy. Our observations revealed that senescent cells exhibited distinct areas of irregularly shaped MAVS aggregates, which were not present in proliferating cells (Figure 4a & b).

To further study the role of MAVS in senescence, we knocked it down using a pool of over 30 siRNAs to maximize knock down efficiency (Figure 4c). RNA sequencing revealed that MAVS knockdown significantly reduced the expression of several SASP components during senescence, underscoring its critical role in this process (Figure 4d). GO enrichment analysis of the top downregulated genes upon MAVS knockdown in senescent cells demonstrated a significant suppression of various inflammatory processes, notably the chronic inflammatory response and regulation of cytokine secretion (Figure 4e). Additionally, similar to the deletion of MDA-5 and RIG-I, MAVS knockdown in senescent cells resulted in a negative enrichment score for the SenMayo and SenSig gene panels (Figure 4f). Importantly, MAVS knockdown did not significantly affect the expression of genes associated with cell proliferation, reinforcing its specific role in the modulation of the SASP (Figure 4g).

### miMOMP is a driver of mtRNA cytosolic leakage in senescence

**Figure 5:**
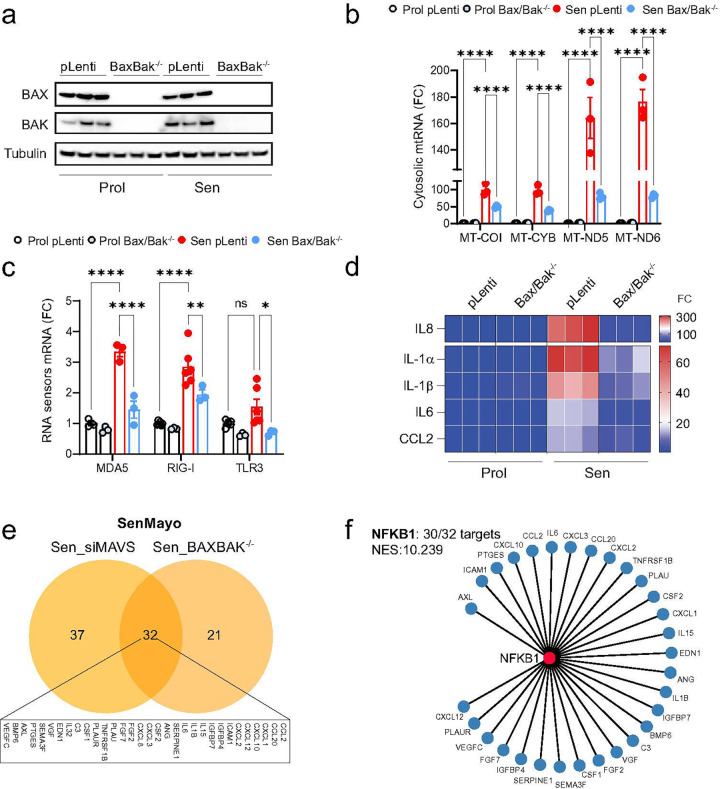
BAX and BAK mediate the leakage of mtRNA into the cytosol of senescent cells. Human fibroblasts deficient in BAX and BAK (BAX−/− BAK−/−) were generated using CRISPR-Cas9 gene editing. (a) Western blot showing successful deletion of BAX and BAK in proliferating and senescent MRC5 fibroblasts. n=3 independent experiments. (b) qPCR quantification of cytosolic mtRNA transcripts in the cytosol of proliferating and senescent BAX−/− BAK−/− cells. (c) mRNA expression of cytosolic RNA sensors in proliferating and senescent BAX−/− BAK−/− cells. (d) mRNA levels of SASP-related genes that are significantly increased in senescence and decreased by deletion of BAX/BAK. n=3 independent experiments. (e) Venn diagram depicting the overlap of commonly downregulated genes between senescent cells lacking MAVS (Sen_siMAVS) and deficient for BAX/BAK (Sen_BAXBAK−/−). (f) Using iRegulon, the transcription factor NF-κB1 was found to control 30 of the 32 overlapping target genes from (e), indicating it is the most likely key regulator (NES 10.239) for these SASP factors. Data are mean ± s.e.m. Statistical significance was assessed by a one-way ANOVA followed by Tukey’s multiple comparison test (b, c).

We have previously shown that the formation of BAX and BAK macropores, a process essential for mitochondrial outer membrane permeabilization (MOMP), in a subset of mitochondria in senescent cells leads to the leakage of mtDNA into the cytosol^17^. To investigate if the same mechanism causes the leakage of mtRNA during senescence, we utilized CRISPR-Cas9 gene editing to generate human fibroblasts deficient in both BAX and BAK (Figure 5a).

Our findings reveal that the combined deletion of BAX and BAK significantly reduced the cytosolic release of mtRNA in senescent cells (Figure 5b). This reduction was accompanied by decreased expression of RNA sensors RIG-I, MDA5, and TLR3 (Figure 5c). Consistent with the role of BAX and BAK in mtRNA release and SASP activation, we confirmed that the deletion of BAX and BAK significantly reduced the expression of several SASP components (Figure 5d). These results suggest that miMOMP, through the formation of BAX and BAK macropores, is a key driver of mtRNA cytosolic leakage in senescent cells, thereby contributing to the activation of the SASP. We then conducted a comparative analysis of global gene expression profiles between senescent cells deficient in BAX and BAK and those with MAVS knockdown. Our investigation of the SenMayo gene set, predominantly consisting of SASP-related genes, revealed that the expression of 32 genes was consistently downregulated following the deletion of BAX and BAK and the knockdown of MAVS (Figure 5e). Further examination demonstrated that 30 of these 32 genes were transcriptionally regulated by NFKB1, a key modulator of the SASP (Figure 5f). To investigate the role of BAX and BAK in senescence *in vivo*, we utilized Bax^fl/fl^ Bak^−/−^ mice and performed tail vein injections with AAV8-TBG-Cre virus to specifically delete BAX in the liver. It is known that cells in Bak^−/−^ mice can still undergo MOMP, while deletion of both Bax and Bak effectively blocks MOMP^29^.

**Figure 6:**
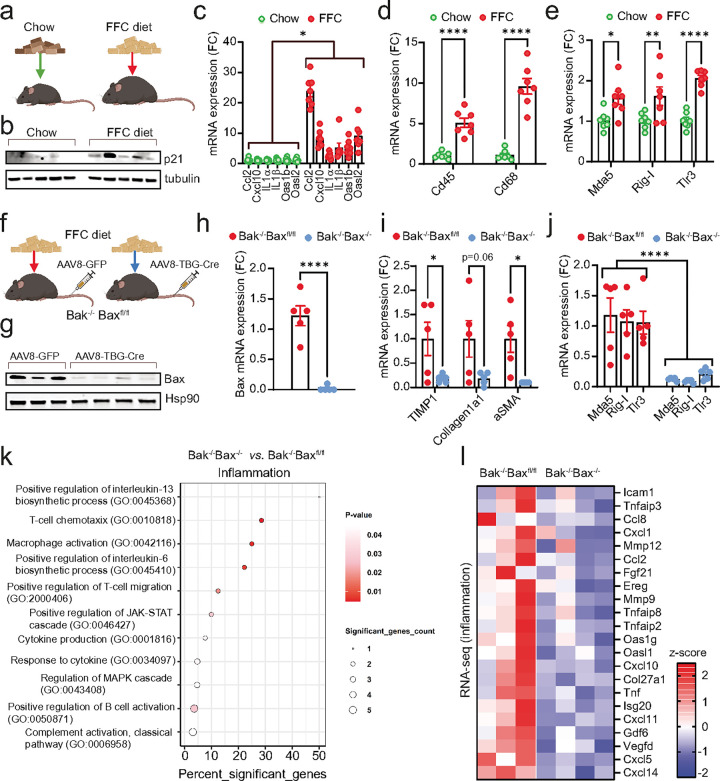
Hepatocyte-specific deletion of BAX/BAK reduces expression of RNA sensors and inflammation in the liver during MASH. (a) Wild-type mice (10–12 weeks old) were fed either a chow or high fat, fructose and cholesterol (FFC) diet for 24 weeks to induce MASH. (b) Western blot showing expression of the senescence marker, p21, in the liver of chow- and FFCfed mice. n=5 mice per group. (c-e) mRNA expression levels of (c) SASP-related genes, (d) immune cell markers, Cd45 and Cd68 and (e) cytosolic RNA sensors in the livers from chow and FFC-fed mice. n=7 mice per group. (f) Bak−/− Baxfl/fl mice (10–12 weeks old) were fed an FFC diet for 16 weeks to induce MASH. Mice were then injected with either AAV8-GFP (control) or AAV8-TBG-Cre virus via the tail vein to induce deletion of Bax specifically in hepatocytes. (g) Western blot showing successful deletion of Bax in the liver following AAV8-TBG-Cre injection. n=3–4 mice per group. (h-j) qPCR quantification of (h) Bax, (i) markers of fibrosis and (j) RNA sensors in livers of FFC-fed Bak−/− Baxfl/fl and Bak−/− Bax−/− mice. n=5 mice per group. (k) Gene ontology (GO) term enrichment analysis showing pathways related to inflammation that are significantly altered between livers from FFC-fed Bak−/− Baxfl/fl and Bak−/− Bax−/− mice. (l) Columnclustered heatmap of inflammation genes that are significantly downregulated in the liver of FFC-fed mice upon deletion of Bax and Bak. Color intensity represents the column z-score. Statistical significance was assessed by a two-sided Student’s unpaired t-test (c-e, h-j). *p<0.05, **p<0.01, ****p<0.0001.

We subjected the mice to a high Fructose, Fat, and Cholesterol (FFC) diet, commonly used as a model of MASH, which has been shown to induce senescence in the liver^30^ (Figure 6a). Consistent with previous findings, the FFC diet led to increased protein expression of p21 (Figure 6b) and elevated mRNA expression of several senescence-associated secretory phenotype (SASP) factors and markers of immune cell infiltration and activation, such as Cd45 and Cd68 in the liver (Figure 6d). Importantly, we also observed increased expression of RNA sensors Rig-I, Mda-5, and Tlr3 in the liver in mice under FFC diet (Figure 6e).

Supporting the hypothesis that Bax and Bak regulate the SASP via RNA sensor expression during diet-induced MASH, we found that hepatocyte-specific deletion of Bax and Bak (Figure 6 f–h) significantly reduced the expression of RNA sensors Rig-I, Mda-5, and Tlr3, as well as inflammatory factors, and markers of fibrosis (Figure 6i–j & **Extended Data Figure 6a**). The deletion of BAX and BAK did not affect body or liver weights and showed no impact on apoptosis markers (**Extended Data Figure 6b-e**). This suggests that their influence on SASP and RNA sensor expression is independent of cell death. To further investigate the impact of liver specific Bax and Bak deletion in the context of MASH, we performed RNA sequencing. This analysis revealed that Bax and Bak deletion significantly altered GO terms associated with inflammatory processes (Figure 6k). Additionally, commonly described SASP factors were significantly downregulated upon Bax and Bak deletion (Figure 6l).

These findings underscore the important role of Bax and Bak in modulating the expression of RNA sensors and the SASP in the context of diet-induced senescence in the liver.

## Discussion

Mitochondrial dysfunction is a hallmark of cellular senescence^31^ and a critical driver of the senescence-associated secretory phenotype (SASP)^15^. While the SASP has been attributed to the activation of DNA sensing pathways such as cGAS-STING^32^ by either nuclear^33,34^ or mitochondrial DNA^17^, the role of mitochondrial RNA (mtRNA) in cellular senescence has not been investigated.

Our study provides new insights into the role of mtRNA as a driver of the SASP. Mitochondrial RNA shares structural similarities with bacterial RNA, such as unmethylated CpG motifs, which are recognized by pattern recognition receptors (PRRs) as pathogen-associated molecular patterns (PAMPs), thereby triggering an immune response^35^. Moreover, mtRNA differs from nuclear mRNA in that it lacks a 5’ end methylated cap^36^. Under normal conditions, mtRNA is confined within mitochondria, and its release into the cytosol is abnormal. However, mtRNA can form double-stranded RNA (dsRNA) structures, and when present in the cytosol, dsRNA is typically associated with viral infections, leading the immune system to recognize it as a danger signal^19,37^.

Our data strongly support that mtRNA accumulates in the cytosol of senescent cells and activates the SASP via RNA sensors RIG-I and MDA5. Furthermore, we demonstrate that inhibition of RIG-I and MDA5 and the mitochondrial antiviral-signaling protein (MAVS) significantly reduces the SASP. Mechanistically, our findings demonstrate that the cytosolic leakage of mtRNA is dependent on the formation of macropores by BAX and BAK in a subset of mitochondria during senescence. Deleting BAX and BAK significantly reduces mtRNA leakage, RNA sensor expression, and the SASP *in vitro* and *in vivo*. Previous work showed that inhibiting mitochondrial outer membrane permeabilization (miMOMP) through pharmacological or genetic deletion of BAX and BAK suppresses the SASP during aging^17^. Our recent data extends this observation to a model of metabolic dysfunction-associated steatohepatitis (MASH), which features increased senescence and inflammation.

These findings highlight that not only cytosolic DNA but also mitochondrial-derived RNA and its downstream signaling pathways are key players in the regulation of the SASP. This discovery highlights the need for further research to understand the relative contributions of different nucleic acid species in the regulation of the SASP and inflammation. Our study opens new avenues for exploring mtRNA as a potential target for modulating the inflammatory responses associated with cellular senescence.

## Material and Methods:

### Cell culture and treatments

Human embryonic lung MRC5 fibroblasts and IMR90 fibroblasts were grown in Dulbecco’s Modified Eagle’s Medium (Sigma, D5796) supplemented with 10% heat-inactivated fetal bovine serum (FBS), 100 units/ml penicillin, 100 μg/ml streptomycin and 2 mM L-glutamine and maintained at 37°C with 5% CO_2_. MRC5 fibroblasts were cultured in atmospheric oxygen conditions and IMR90 fibroblasts were cultured under low oxygen (3%) conditions.

HEK293T cells were used for lentiviral transduction and were cultured in DMEM as described above and further supplemented with 1% non-essential amino acids (Sigma, M7145), 500μl/μl G418 antibiotic (Sigma, A1720) and 1 mM sodium pyruvate (Sigma, S8636).

Stress-induced senescence was achieved by exposing cells to X-ray irradiation at 10 Gy (MAFs) or 20 Gy (human fibroblasts). Replicative senescence was achieved by serially passaging cells until they reached their replicative potential and performed less than 0.5 population doublings for at least 4 weeks. For chemotherapy-induced senescence, cells were treated with either 250 nM of doxorubicin or 50μM of etoposide for 24 hours and harvested at day 10 or day 8 post-treatment, respectively. Senescence was confirmed by presence of p16 and p21, and absence of proliferation markers Ki67 or EdU incorporation.

For inhibition of mitochondrial RNA polymerase (POLRMT), cells were exposed to 20 Gy X-ray irradiation and treated with 1μM of the inhibitor IMT-1 (MedChem, HY-134539) for 10 days. Treatment was refreshed every other day.

For TLR3 inhibition, cells were exposed to 20 Gy X-ray irradiation and treated with 50 μM of TLR3/dsRNA complex inhibitor (Sigma, 614310) for 10 days. Treatment was refreshed every day for the duration of the experiment.

### Parkin-mediated mitochondria clearance

Parkin-mediated widespread mitochondrial clearance was performed as in^14,15^. Briefly, proliferating, or irradiated Parkin-expressing IMR90 fibroblasts were treated with 12.5 μM CCCP (Sigma, C2759) (3 days after irradiation) for 48 hours (CCCP was refreshed every 24 hours). Mitochondria-depleted cells were then transfected with isolated mitochondrial RNA (as described below) at 8 days post-irradiation and harvested at 10 days after irradiation.

### Mitochondrial RNA transfection

Proliferating MRC5 fibroblasts and Parkin-expressing IMR90 fibroblasts were transfected with 2.5 μg of isolated mitochondrial RNA using Lipofectamine MessengerMax Reagent (Invitrogen,

LMRNA003), according to the manufacturer’s instructions. Cells were incubated with mtRNA for 48 hours and then harvested for analysis.

### Subcellular fractionation

For cytosolic fraction analysis, subcellular fractionation was performed using Subcellular Protein Fractionation Kit for Cultured Cells (Thermo Fisher, 78840), according to manufacturer’s instructions.

### Mitochondrial isolation and mitochondrial RNA extraction

For the mitochondrial-enriched fraction, followed by a rinse in ice-cold PBS, cells were collected by scraping the flask with 5 ml of ice-cold PBS. Cells were centrifuged at 800*g* for 5 min at 4 °C and resuspended in mitochondrial isolation solution (MIS) (20 mM HEPES-KOH pH 7, 220 mM mannitol, 70 mM sucrose, 1 mM EDTA, 0.5 mM PMSF, 2 mM DTT). The samples were transferred to a glass homogenizer and cells were broken open using 60 strokes. The homogenate was centrifuged at 800*g* for 5 min at 4 °C. The supernatant was further centrifuged at 800*g* for 5 min at 4 °C. An aliquot of the supernatant was collected and stored as the whole-cell extract. The remaining was centrifuged at 16,100*g* for 10 min at 4 °C. The supernatant was collected as the cytosolic fraction. The pellet containing mitochondria was resuspended in 1 ml of MIS and centrifuged again at 16,100*g* for 10 min at 4 °C. This step was repeated, and the resulting pellet was resuspended in 100 μl of MIS. For mitochondrial RNA extraction, the mitochondrial pellet was centrifuged at 16,100*g* for 10 min at 4 °C, and RNA extraction was performed using the QIAGEN RNeasy Mini Kit (Qiagen, 74106) according to manufacturer’s instructions.

### CRISPR/CAS9-based genome editing

The following plasmids were used: LentiCRISPR v2 hBAK (Addgene, 129579), LentiCRISPR v2 hBAX (Addgene, 129580), LentiCRISPR v2-puro (Addgene, 52961), pLV[CRISPR]-hCas9:T2A:Puro-U6>hDDX58[gRNA#3708] (VectorBuilder, VB900079–3851tta) and pLV[CRISPR]-hCas9:T2A:Puro-U6>hIFIH1[gRNA#141] (VectorBuilder, VB900098–8309keh).

For lentiviral transduction, HEK293FT cells were transfected with the plasmids above together with the packaging and envelope plasmids VSVG and Gag-Pol (Sigma) using Lipofectamine 3000 (Invitrogen, L3000015) according to the manufacturer’s instructions. Two days later, the supernatant from the transfected HEK293FT cells containing viral particles was filtered using a 0.45 μm pore PVDF filter, mixed with 10 μg/ml polybrene and used to infect the cells of interest. Following infection, cells were selected for successful CRISPR/Cas9 deletion by using 1 μg/ml Puromycin.

### siRNA gene knockdown

MRC5 fibroblasts were transiently transfected with 30nM of a pool of siRNAs against MAVS or negative control (Galen Molecular, si-K005-ABC123). Cells were transfected using DharmaFECT 2 transfection reagent (Thermo Fisher, T-2002–03) following manufacturer’s instructions. For senescent cells, cells were transfected one day prior to X-ray irradiation and then again on day 7 post-irradiation. Cells were harvested at day 10 following irradiation for analysis.

### qPCR

RNA was extracted using the RNAeasy Mini Kit (Qiagen, 74106) according to the manufacturer’s instructions. Complementary DNAs were synthesized using the High-Capacity cDNA Reverse Transcription Kit (Thermo Fisher Scientific, 4368814) according to the manufacturer’s instructions. qPCR was performed using ToughMix Perfecta (PerfeCTa qPCR ToughMix, QuantaBio, 95112–250) using the CFX96TM Real-Time System (Bio-Rad). mRNA levels were calculated using the 2−ΔΔCt method and normalized to a housekeeping gene. DNA was quantified using the Nanodrop and stored at −20 °C. The primers used are listed in Supplemental Table 1.

### Western blotting

Cells were lysed in lysis buffer (150 mM NaCl, 1% NP40, 0.5% sodium deoxycholate, 0.1% SDS, 50 mM Tris pH 7.4, 1× phosphatase and protease inhibitors cocktail in H_2_O) and the protein concentration was determined using the Bio-Rad protein assay (Bio-Rad, reagent A, 500–0113; reagent B, 500–0114; reagent C, 500–0115). Equal amounts of protein (at least 15 μg) from each sample were resolved on Tris-glycine gels and samples were then blotted onto a 0.45 μm polyvinylidene difluoride (PVDF) membrane (Millipore) using Trans-Blot SD Semi-Dry Transfer Cells (Bio-Rad). Membranes were blocked with PBS-Tween blocking buffer (5% milk powder, 0.05% Tween-20 in PBS) and then incubated with primary antibodies at 4 °C overnight (a list of the antibodies used is provided in Supplementary Table 2). After washes in distilled water, the membranes were incubated with a peroxidase-conjugated secondary antibody for 1 h at room temperature. The membranes were then incubated with either Clarity ECL Western Blot Substrate (Bio-Ras, 170–5060) or the KwikQuant Western blot detection kit (Kindle Bioscience, R1100) according to manufacturer’s instructions, and visualized using iBright 1500 (Invitrogen). The antibodies used are listed in Supplemental Table 2.

### RNA Immunoprecipitation (RIP) Assay

RNA immunoprecipitation (RIP) assay was performed using the Magna RIP RNA-Binding Protein Immunoprecipitation Kit (Merck Millipore, MA, USA) according to manufacturer’s directions. Briefly, 8 × 10^6^ MRC5 cells (proliferating and senescent) were scraped from T175 flasks, centrifuged at 1500 RPM for 5 minutes and washed in cold 1X PBS. The pellets were then resuspended in 200 μl of RIPA cell lysis buffer. The Magnetic Beads Protein A/G (CS203178; Merck Millipore, MA, USA) were suspended in RIPA cell lysis buffer, bound to a magnetic separator and washed with RIPA wash buffer and again bound to the magnetic separator with the supernatant being removed. The magnetic beads were resuspended in 100 μl of RIPA wash buffer. 5 μg of RIG-I (20566–1-AP; Proteintech, IL, USA) or MDA5 (21775–1-AP; Proteintech, IL, USA) or rabbit IgG antibody were preincubated with beads, washed, and resuspended in 900 μl of RIP Immunoprecipitation buffer including EDTA and RNAse inhibitor. RIP lysate was centrifuge at 14,000 RPM for 10 minutes at 4°C. 100 μL of the supernatant was added to each beads-antibody complex in RIP Immunoprecipitation Buffer and incubated overnight rotating at 4°C. The following day, the lysate-beads-antibody complex was washed 5X in cold RIP wash buffer. Following the final wash step, the lysate-beads-antibody complex was incubated in the Proteinase K buffer at 55^o^C for 30 minutes. The tubes were placed on the magnetic separator and the supernatant was removed followed by addition of 250 μl of RIP buffer. RNA was subsequently isolated using TRIzol^™^ Reagent (Invitrogen, Carlsbad, CA) according to manufacturer’s directions. RNA was reverse transcribed using SuperScript III First-Strand synthesis (Invitrogen, Carlsbad, CA) and qPCR for mtRNA transcripts was performed.

### RNA-sequencing

Sequencing libraries were made from poly-A RNA, as recommended by Illumina, and sequenced using either an Illumina GAIIX or a NextSeq 500 sequencer. RNA-seq paired-end reads were assessed for quality using the ‘FastQC’ algorithm, then aligned to the human genome using the splice-aware aligner STAR with a two-pass alignment pipeline. Reference splice junctions were provided by a reference transcriptome from the Gencode GRCh38 (hg38) build. BigWig files were generated using DeepTools. Raw read counts per gene were calculated using htseq-count. The read count matrix was then used for differential expression analysis with the linear modeling tool DESeq2. Significantly changing expression was defined as an FDR-corrected p-value ≤0.05. FPKM (Fragments Per Kilobase of transcript per Million mapped reads) values were generated using Cufflinks. Gene ontology analysis was performed using Gene Set enrichment Analysis (GSEA) and Ingenuity^®^ Pathway Analysis (IPA) software. Transcription factor analysis was created iRegulon in Cytoscape. A gene-based motif collection (1120 ChIP-seq tracks, regulatory region 20kb centered around TSS, ROC threshold for AUC: 0.03, Rank threshold 5.000) was used for the analysis.

### Immunocytochemistry

Cells grown on coverslips were fixed using 3.7% paraformaldehyde in DMEM for 10 minutes. Cells were washed in PBS and then permeabilized in 0.1% Triton X-100 in PBS for 10 minutes at room temperature. Following blocking in 1% bovine serum albumin (BSA) in PBS-Tween20 for 1 hour, cells were then with primary antibody overnight at 4°C (antibodies diluted in 1% BSA/PBS). The following day, cells were washed three times in PBS-Tween20, and secondary antibody was applied and incubated for 45 minutes at room temperature. Coverslips were mounted onto glass microscope slides with ProLong Gold Antifade Mountant with DAPI (Invitrogen).

For MAVS, cells were fixed in 0.05% glutaraldehyde in 4% paraformaldehyde for 10 minutes.

The antibodies used are listed in Supplemental Table 3.

### Terminal deoxynucleotidyl transferase-mediated deoxyuridine triphosphate nick-end labeling (TUNEL) assay

Apoptosis in tissue was measured by the fluorescent terminal deoxynucleotidyl transferase-mediated deoxyuridine triphosphate nick-end labeling (TUNEL) assay on frozen liver sections. Liver tissue samples were cryopreserved in optimum cutting temperature compound (Takeda) immediately after harvest. Tissue sections were cut at 7 μm on a cryomicrotome (Leica) and stored at −80°C before use. The TUNEL assay was then performed using the manufacturer’s protocol (#11684795910, Roche). Apoptotic cells were quantified by counting TUNEL-positive nuclei per high-power field in 20 random microscopic fields using a fluorescent microscope (Eclipse 80i; Nikon) in a blinded manner.

### Microscopy

Imaging was performed using widefield microscope (DMi8 Leica) and super-resolution microscope (confocal microscopy using the AiryScan type detector LSM980 Zeiss AiryScan).

Analysis of cytosolic mitochondrial RNA and MAVS aggregation was performed using ImageJ.

### Mouse models

All animal experiments were performed according to protocols approved by the Institutional Animal Care and Use Committee (IACUC) at Mayo Clinic. Mice were kept at the Mayo Clinic Rochester animal facility with a 12-hour light-dark cycle. All mice were placed on a diet at 10–12 weeks of age. The chow diet was a standard rodent diet (PicoLab 5053, LabDiet) with tap water. FFC diet contains high fat (40% calories) and high cholesterol (0.2%) (#AIN-76A Western Diet; TestDiet), with fructose (18.9 g/L) and glucose (23.1 g/L) added to the drinking water (total sugar 42 g/L) as previously described (PMID: 37267252). For characterizing senescence and expression of RNA sensors in the liver, C57Bl6/J male mice (purchased from the Jackson Laboratory) were placed on a chow or FFC diet for 24 weeks. For Bak and Bax studies, 10–12 week-old Bak^−/−^ Bax^fl/fl^ male mice (obtained from the Jackson Laboratory, strain #006329) were injected via tail vein with 1.5 × 10^11^ GC/mouse of either AAV8-TBG-Cre (for hepatocyte-specific deletion) or with control virus (AAV8-GFP), both obtained from Vector Builder. Two weeks post-injection, mice were placed into a thermoneutral caging system (Solace Zone) and were kept on the FFC diet for 16 weeks. At the end of the feeding period, mice were sacrificed under general anesthesia induced by a combination of xylazine and ketamine. The liver was harvested and processed for further examination.

## Data Availability

The RNA-seq datasets generated and analyzed during the current study are available in the GEO repository.
